# Serotonin transporter gene *(SLC6A4)* polymorphism and susceptibility to a home-visiting maternal-infant attachment intervention delivered by community health workers in South Africa: Reanalysis of a randomized controlled trial

**DOI:** 10.1371/journal.pmed.1002237

**Published:** 2017-02-28

**Authors:** Barak Morgan, Robert Kumsta, Pasco Fearon, Dirk Moser, Sarah Skeen, Peter Cooper, Lynne Murray, Greg Moran, Mark Tomlinson

**Affiliations:** 1 Global Risk Governance Programme, Institute for Safety Goverance and Criminology, Law Faculty, University of Cape Town, Rondebosch, South Africa; 2 NRF Centre of Excellence in Human Development, DVC Research Office, University of Witwatersrand, Johannesburg, South Africa; 3 Neonatal Unit, Department of Women’s and Children’s Health, Karolinska Institute, Stockholm, Sweden; 4 Department of Genetic Psychology, Faculty of Psychology, Ruhr University Bochum, Bochum, Germany; 5 Research Department of Clinical, Educational and Health Psychology, Faculty of Brain Sciences, University College London, London, United Kingdom; 6 Department of Psychology, Stellenbosch University, Stellenbosch, South Africa; 7 School of Psychology and Clinical Language Sciences, University of Reading, Reading, United Kingdom; 8 Department of Psychology, University of Cape Town, Rondebosch, South Africa; 9 Department of Psychology, Western University, London, Ontario, Canada; Makerere University Medical School, UGANDA

## Abstract

**Background:**

Clear recognition of the damaging effects of poverty on early childhood development has fueled an interest in interventions aimed at mitigating these harmful consequences. Psychosocial interventions aimed at alleviating the negative impacts of poverty on children are frequently shown to be of benefit, but effect sizes are typically small to moderate. However, averaging outcomes over an entire sample, as is typically done, could underestimate efficacy because weaker effects on less susceptible individuals would dilute estimation of effects on those more disposed to respond. This study investigates whether a genetic polymorphism of the serotonin transporter gene moderates susceptibility to a psychosocial intervention.

**Methods and findings:**

We reanalyzed data from a randomized controlled trial of a home-visiting program delivered by community health workers in a black, isiXhosa-speaking population in Khayelitsha, South Africa. The intervention, designed to enhance maternal-infant attachment, began in the third trimester and continued until 6 mo postpartum. Implemented between April 1999 and February 2003, the intervention comprised 16 home visits delivered to 220 mother–infant dyads by specially trained community health workers. A control group of 229 mother–infant dyads did not receive the intervention. Security of maternal-infant attachment was the main outcome measured at infant age 18 mo. Compared to controls, infants in the intervention group were significantly more likely to be securely attached to their primary caregiver (odds ratio [OR] = 1.7, *p* = 0.029, 95% CI [1.06, 2.76], *d* = 0.29). After the trial, 162 intervention and 172 control group children were reenrolled in a follow-up study at 13 y of age (December 2012–June 2014). At this time, DNA collected from 279 children (134 intervention and 145 control) was genotyped for a common serotonin transporter polymorphism. There were both genetic data and attachment security data for 220 children (110 intervention and 110 control), of whom 40% (44 intervention and 45 control) carried at least one short allele of the serotonin transporter gene. For these 220 individuals, carrying at least one short allele of the serotonin transporter gene was associated with a 26% higher rate of attachment security relative to controls (OR = 3.86, *p* = 0.008, 95% CI [1.42, 10.51], *d* = 0.75), whereas there was a negligible (1%) difference in security between intervention and control group individuals carrying only the long allele (OR = 0.95, *p* = 0.89, 95% CI [0.45, 2.01], *d* = 0.03). Expressed in terms of absolute risk, for those with the short allele, the probability of secure attachment being observed in the intervention group was 84% (95% CI [73%, 95%]), compared to 58% (95% CI [43%, 72%]) in the control group. For those with two copies of the long allele, 70% (95% CI [59%, 81%]) were secure in the intervention group, compared to 71% (95% CI [60%, 82%]) of infants in the control group. Controlling for sex, maternal genotype, and indices of socioeconomic adversity (housing, employment, education, electricity, water) did not change these results. A limitation of this study is that we were only able to reenroll 49% of the original sample randomized to the intervention and control conditions. Attribution of the primary outcome to causal effects of intervention in the present subsample should therefore be treated with caution.

**Conclusions:**

When infant genotype for serotonin transporter polymorphism was taken into account, the effect size of a maternal-infant attachment intervention targeting impoverished pregnant women increased more than 2.5-fold when only short allele carriers were considered (from *d* = 0.29 for all individuals irrespective of genotype to *d* = 0.75) and decreased 10-fold when only those carrying two copies of the long allele were considered (from *d* = 0.29 for all individuals to *d* = 0.03). Genetic differential susceptibility means that averaging across all participants is a misleading index of efficacy. The study raises questions about how policy-makers deal with the challenge of balancing equity (equal treatment for all) and efficacy (treating only those whose genes render them likely to benefit) when implementing psychosocial interventions.

**Trial Registration:**

Current Controlled Trials ISRCTN25664149

## Abstract 2

In light of genetic information Barak Morgan and colleagues re-analyze data from a randomized controlled trial to explore the genetic susceptibility to a psychosocial intervention.

## Author summary

**Why was this study done?:**

- It has been shown that individuals with the short form of a gene involved in nerve signaling in the brain are generally sensitive, or “susceptible,” to psychosocial interventions (i.e., they benefit), whereas individuals with the long form of the gene are insensitive, or “nonsusceptible,” deriving little benefit from the same intervention.
- This study was conducted to investigate whether such a gene × intervention interaction was present in a previously conducted randomized controlled home-visiting intervention trial designed to increase levels of secure maternal-infant attachment at 18 mo of age in a population in South Africa.

**What did the researchers do and find?:**

- Genotyping revealed that 40% of the study sample (*n* = 89) carried at least one short allele of the serotonin transporter gene, while 60% of the sample (*n* = 131) carried two long alleles of this gene.
- We reanalyzed the original maternal-infant attachment results and found that the increase in secure attachment found for the intervention in the original trial (odds ratio = 1.7, effect size *d* = 0.29) was almost entirely attributable to children with the short form of the gene (odds ratio = 3.86, effect size *d* = 0.75). For children not carrying the short allele, there was no significant increase in secure attachment with the intervention (odds ratio = 0.95, effect size *d* = 0.03).
- Children carrying the short form of the gene whose mothers received the intervention were 3.86 times more likely to be securely attached at 18 mo (84% were secure) than children carrying the short form whose mothers did not receive the intervention (58% were secure).
- Among children without the short form, the number who were securely attached did not differ based on whether their mothers received the intervention (71% were secure) or not (70% were secure).

**What do these findings mean?:**

- These results indicate the importance of genetic differences when considering the efficacy of psychosocial interventions aimed at improving developmental outcomes in children living in the context of socioeconomic adversity.
- The findings also raise complex questions regarding spending limited resources implementing costly interventions on individuals who may not benefit because of their genetic makeup and who may comprise the majority of the population (60% in this case), but where these same interventions may nevertheless confer large benefit for a subgroup of genetically susceptible individuals (40% in this case).

## Introduction

Early childhood development is increasingly recognized as a public health priority that requires attention and investment, and specific targets and indicators addressing this area are included in the recent Sustainable Development Goal (SDG) framework and the United Nations Secretary General’s Global Strategy for Women’s, Children’s and Adolescents’ Health [1,2]. With the knowledge that more than 250 million children younger than 5 y globally will fail to reach their full developmental potential [3], clear recognition of the damaging effects of poverty on early childhood development has fueled an interest in psychosocial interventions aimed at mitigating these harmful consequences in order to promote lifelong health and prosperity.

Gains in child development have been generated by psychosocial interventions to improve child nutrition and development and to address the mental health of caregivers [4]. Interventions in the early years are cost-effective [5], can reduce inequity [6], and have been shown to have an impact on adult health outcomes [7]. Parenting programs focused on early child development are a good example of a delivery mechanism for the prevention and reduction of childhood disadvantage [8]. However, the efficacy of early child development interventions has, for the most part, been quantified by averaging individual outcomes across an entire sample and then, to varying extents, controlling for other factors, mostly of an extrinsic nature (e.g., maternal, environmental, and demographic factors). However, if individual differences in susceptibility to intervention are not considered (e.g., temperament, biological stress reactivity, and genetic differences), averaging of outcomes could lead to a misleading assessment of efficacy, because weaker effects on less susceptible individuals would dilute the estimation of effects on those more liable to respond [9,10]. In order to optimize the benefit of interventions, accurate evaluation of efficacy—including for whom the intervention does and does not work—is essential.

In 2003, Caspi et al. [11] first suggested that there were genetic differences in susceptibility to environmental influence in relation to depression. They reported observational findings indicative of a gene × environment (G×E) interaction. When individuals had experienced childhood maltreatment or stressful life events in early adulthood, carriers of the short allele (short/short or short/long genotype) of a length polymorphism in the regulatory region of the serotonin transporter gene (termed the *5HTT*-linked polymorphic region [5HTTLPR]) were more liable than carriers of two long alleles (long/long genotype) to suffer from depression [11]. Subsequent studies have continued to yield evidence of G×E interaction at the 5HTTLPR locus [12].

While this evidence is important, only recently have investigators begun to make use of interventions where participants are randomly allocated to different environmental conditions to test the causal status of G×E interactions. Randomized controlled trials (RCTs) have been estimated to have 13 times the statistical power of G×E studies in which individuals (and therefore genotypes) are not randomly assigned to categorically different environments (e.g., intervention and no intervention) [10]. RCTs have, inter alia, two other big advantages: precluding gene–environment correlations (where genes and environments “choose” one another) and overcoming many of the problems with accurately measuring the environment of interest (e.g., maltreatment of children) [10]. A recent meta-analysis of 11 field RCTs investigated gene × intervention (G×I) interactions involving several genes thought to confer greater susceptibility to interventions, including 5HTTLPR [9]. The meta-analysis showed that intervention benefits were significantly stronger in those with susceptible genotypes, with an overall odds ratio (OR) of 3.17 (*p* < 0.01) for individuals with susceptible genotypes, compared to only 1.16 (*p* = 0.6) among individuals with nonsusceptible genotypes. Although striking, this meta-analytic evidence of genetic differential susceptibility to intervention rests upon an empirical base beset by several limitations. These include reliance on small sample sizes and special populations (e.g., maltreated children [13–15] or stroke patients [16]), mixed results for some genes [9], lack of ethnic and socioeconomic diversity (mostly middle-class white individuals) [13], and inconsistencies across different ethnic groups [17]. Crucially, given the interest in tackling poverty through early child development interventions in low- and middle-income countries (LMICs), it is striking that, to the best of our knowledge, no study has investigated differential susceptibility to intervention outside the US and Europe.

Security of attachment, which can be objectively and reliably assessed in infancy [18], is an important indicator of positive early socio-emotional development [19–21]. A range of studies have shown that, compared to insecure attachment, secure attachment is associated with better subsequent outcomes, including reduced externalizing behavior problems and better social competence [22,23]. There is also emerging evidence that secure infant attachment and the sensitive maternal care that promotes it are linked to better growth, physical health, and cognitive development [19–21,24]. Promoting secure mother–infant attachment is therefore an important focus for prevention studies, and, indeed, a wide range of interventions have been developed that appear to be successful in promoting secure attachment [25]. The majority of these interventions target the responsiveness of the mother’s caregiving behavior in relation to the infant’s attachment cues and communications, and have been delivered as primary or secondary prevention in a wide range of contexts within high-income countries.

Here, we report the results of a reanalysis of data from a RCT in which we test for a G×I interaction in early child development. Our study focuses on 5HTTLPR as a potential genetic moderator of the efficacy of a home-visiting intervention that was designed to improve attachment in mother–infant dyads in an impoverished isiXhosa-speaking community in South Africa. The intervention, known as Thula Sana (“hush baby” in isiXhosa), was a manualized home-visiting parenting program that aimed to promote security of infant attachment (the primary outcome of the original trial) by enhancing maternal sensitivity to infant characteristics and communication and by supporting management of infant distress [26,27].

This intervention was evaluated in an individually randomized controlled trial over a 4-y period, beginning in 1999, with a sample of 449 pregnant women. At infant age 18 mo, infant attachment status was assessed using standardized laboratory procedures [26]. Compared to controls who received no intervention, infants in the intervention group were significantly more likely to be securely attached to their primary caregiver (OR = 1.7, *p* = 0.029, 95% CI [1.06, 2.76]) [26]. This result equates to an effect size of Cohen’s *d* = 0.29, consistent with previous reports of modest effect size estimates for such interventions [4]. Further results on maternal sensitivity and maternal depression are reported in Cooper et al. [26]. However, this first report of the trial did not take into account the issue of differential susceptibility, and it is therefore possible that differences in efficacy for susceptible and nonsusceptible individuals may have been overlooked.

A follow-up study of the original Thula Sana cohort at 13 y of age provided the opportunity to address the possibility of genetic differential susceptibility by collecting DNA from both children and mothers. We focused on 5HTTLPR, the polymorphism most frequently investigated with respect to attachment outcomes and related processes in G×E studies [13,28–31]. To date, all studies implicating 5HTTLPR in genetic differential susceptibility to environment for attachment outcomes have been observational and have yielded mixed results (for reviews, see [17,32]). The current study circumvented the inherent limitations of observational research designs [9–11] by testing for a G×I interaction between 5HTTLPR and a home-visiting intervention on attachment security. On the basis of previous studies implicating the short 5HTTLPR allele as the “susceptibility allele” [11,17], genetic differential susceptibility to the intervention was predicted to be high for children carrying at least one short allele and low for children carrying two long alleles.

## Methods

### Ethics

The Health Research Ethics Committee of Stellenbosch University approved this study (Ethics Reference #S12/04/113). Adult caregivers provided written consent for their and their child’s participation, and adolescents signed assent forms prior to participating in the study.

### Design

This is a reanalysis of results from the original Thula Sana RCT. In the original trial, mothers were randomized during pregnancy to receive the Thula Sana intervention or usual care during pregnancy and the first 6 mo after birth. The primary outcomes of the original trial were maternal sensitivity and infant attachment security. The aim of this investigation was to test whether 5HTTLPR genotype moderated the intervention effect on infant attachment security measured at 18 mo. This report presents a reanalysis of the original trial’s primary outcome using genetic information gathered at 13 y.

### Participants

Between April 1999 and February 2003, pregnant mothers from a racially and ethnically homogeneous black, isiXhosa-speaking population inhabiting two geographical areas within Khayelitsha were enrolled in the original Thula Sana study [26]. We made efforts to identify and recruit women who were in their last trimester of their pregnancy (on the basis of the accounts of their gestation that women had received from antenatal clinics). Throughout the recruitment period, over 22 mo, a research assistant regularly visited all the homes door-to-door in both areas to inquire whether anyone had become pregnant or a pregnant woman had moved into the area, and to invite identified women to participate in the study. We identified a consecutive series of 452 women as pregnant within the study area and invited them to take part in the study. Of these, three refused to participate. We then assigned the remaining 449 women to the intervention or control group using minimization, balancing for antenatal depression, whether or not the pregnancy was planned, and which area within Khayelitsha they lived in. In the original trial, they were assessed antenatally and at 2 mo, 6 mo, 12 mo, and 18 mo after birth. The maternal socioeconomic profile for this sample at the time of antenatal interview was as follows: 85% lived in informal housing (shacks), 89% had no formal employment, 44% had no electricity, and 39% had no running water in their home [26]. Later, from December 2012 to June 2014, we enrolled the sample for a long-term 13-y follow-up.

### The intervention

Lay community health workers, themselves all mothers, were selected from the local community, underwent an 8-wk training on delivering the intervention, and were given weekly support and supervision throughout the intervention period. The intervention began in the last trimester of pregnancy, and continued until 6 mo postpartum, during which a total of 16 visits of 1 h each were delivered [26]. The intervention was designed to be suitable for routine delivery within low-resource settings. The content was based closely on *The Social Baby* [33], but it also incorporated the key principles of the World Health Organization’s report *Improving the Psychosocial Development of Children* [34] and the use of items from the Neonatal Behavioral Assessment Scale [35], to sensitize the mother to her infant’s individual capacities and needs. Women in the control group received standard services provided by the local infant clinic as well as fortnightly home visits by a community health worker who assessed the physical and medical progress of mothers and infants (women in the intervention group received these services as well).

### Procedures

Full data collection procedures for the early trial were reported in the first outcome paper [26]. In the follow-up study, children and their caregiver were assessed at the Prevention Research for Community, Family and Child Health study center located in Khayelitsha for approximately 4 h. Only limited and out-of-date address information was available from the original study, and many of the names of areas and roads in the informal parts of Khayelitsha had changed in the period between the original study and the reenrollment period. In addition to going door-to-door to find participants at their old addresses, reenrollment strategies also included engaging local community structures. Most participants were still resident in the area, but one-quarter had migrated to other parts of the country since the infant age 18 mo assessment, with participants located in five different provinces of the country. Wherever possible, the team arranged for these child and mother participants to travel to Cape Town. However, there was a small subgroup of participants who were not able to travel to Cape Town. In these cases, a data collection team travelled to their homes for assessment purposes. At the time of assessment, saliva for DNA extraction was collected from children and whenever possible from their biological mothers as well.

### Measures

#### Maternal-infant attachment

At 18 mo of age, infant attachment status was assessed in 76% of the original 449 mother–infant pairs using the standardized strange situation procedure (SSP) developed by Ainsworth and colleagues [18,26], and used extensively in research in both high-income countries and LMICs [36,37]. To date, the attachment status of children in LMICs has received little research attention, but the construct of attachment and the SSP have been shown to be valid cross-culturally [37]. To the best of our knowledge, there have been only two previous studies assessing attachment in Africa using the procedure [24,38].

At 18 mo, the primary caregiver who participated in the assessment was in all cases the biological mother. To conduct the SSP, the infant was filmed through a one-way mirror in an unfamiliar playroom over a 21-min period divided into seven 3-min episodes, including two episodes of separation and reunion with the mother. M. T. rated the video tapes for security of infant attachment, having been trained to criterion by an established US training program. He used the ABCD coding system to rate infants as secure (B) or as one of three categories of insecure (A, avoidant; C, anxious-resistant; or D, disorganized). These ratings were made blind to all other information about the infants and their mothers. Reliability was confirmed by assessing agreement between M. T. and a second trained UK rater on 16 tapes (four-way κ = 0.96). In the original trial, a total of 318 infants were successfully assessed in the SSP, 265 of whom were amongst the 334 children reassessed at 13 y of age. Of these 265, 40 had been classified as avoidant, 182 secure, 21 resistant, and 22 disorganized. In keeping with the literature and in order to maximize cell sizes in the analysis, we restricted our analyses to the binary distinction between secure and insecure classifications, where insecure included all three insecure categories (A/C/D) pooled together.

#### Genotyping

At the 13-y follow-up, for noninvasive collection of high-quality DNA, 2 ml of saliva was collected by trained and supervised data collectors using Oragene DNA OG-500 (DNA Genotek) saliva self-collection kits at the research center or at participants’ homes. Oragene kits were stored at room temperature and shipped to Germany for molecular genetic analysis. DNA was extracted from saliva samples and purified according to the kit protocol. All samples passed initial quality control, with OD260/OD280 ratios between 1.6 and 2.0. Participants were genotyped for the 43-bp insertion/deletion polymorphism in the regulatory promoter region of the serotonin transporter gene (5HTTLPR) with a standard PCR procedure, as previously described [39]. There was no deviation from Hardy–Weinberg equilibrium (χ^2^ = 1.13, *p* = 0.29).

### Data analysis

The current report tested a single a priori hypothesis that 5HTTLPR genotype, operationalized as the presence versus absence of the short form of 5HTTLPR, would moderate the intervention effect on the primary outcome (secure versus insecure attachment). Security of attachment cannot be measured in children under 11 mo, and therefore it was measured only at the post-intervention follow-up when the infants were 18 mo of age. The analysis was therefore a single-level (i.e., not repeated measure) logistic regression, with the hypothesized moderating effect specified as an intervention group × 5HTTLPR genotype interaction. The primary analysis was conducted without adjustment for covariates, but sensitivity analyses were also conducted, adjusting for covariates; in these analyses we also assessed the impact of missing data (for individuals without both attachment and genetic data, including all individuals lost to follow-up) using multiple imputation, as recommended by a reviewer. We used the fully conditional specification approach to multiple imputation, which is a highly flexible approach capable of accounting for nonlinearity in the relationship between covariates and outcome and which fits an imputation model that is consistent with the substantive model (i.e., explicitly includes the gene and intervention main effects and interaction). Multiple imputations included all model variables, maternal 5HTTLPR genotype, and the only baseline measures that were associated with missingness (time to entry into the trial from start of recruitment and whether the house had electricity and water). Imputation was conducted using the package smcfcs [40] and Stata’s MI procedure based on 100 imputed samples. Details of the imputation are provided in S1 Text and S2 Data.

## Results

### Sample

**Fig 1:**
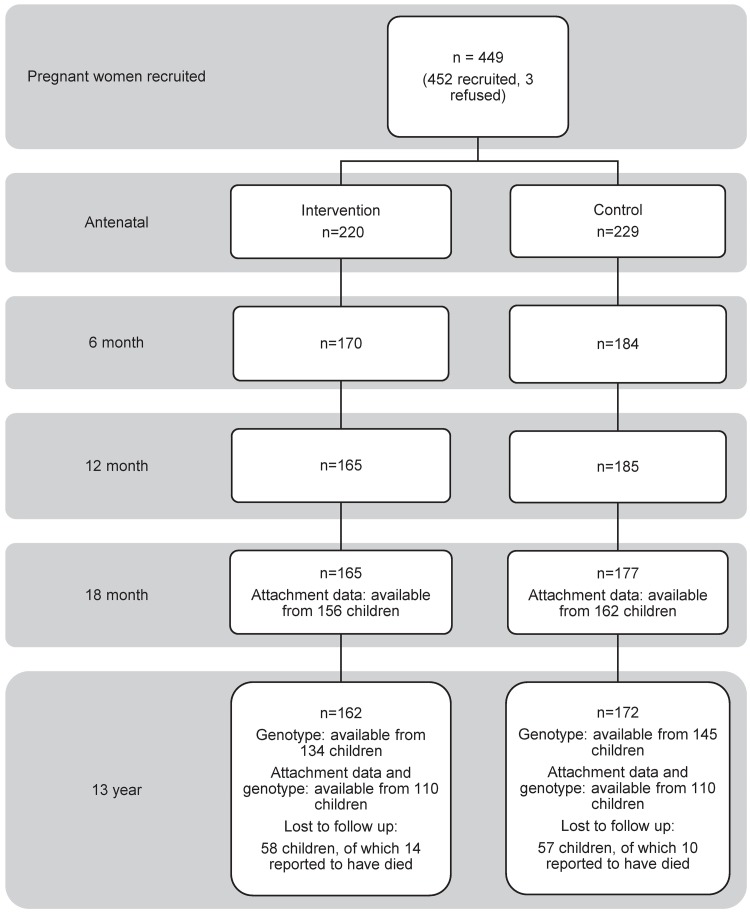
Flowchart showing how the sample of 220 13-y-old children with both 5HTTLPR genotype and attachment security data was assembled for this study.

From December 2012 to June 2014, we reenrolled 334 (74.1%) of the children (162 intervention, 172 control; 166 males, 168 females) from the original sample of 449 mother–child pairs. At 13 y of age, 115 of the original 449 children were lost to follow-up. Of these children, 24 had died since the original randomization process. The remaining 91 could not be contacted. Derivation of the sample used in this study is depicted in Fig 1.

Of the 334 adolescent participants at 13-y follow-up, 279 (131 males, 148 females; 134 intervention, 145 control) provided DNA samples, all of which yielded 5HTTLPR genotype results. There were 220 (104 males, 116 females; 110 intervention, 110 control) adolescents for whom there were both 5HTTLPR genotype and attachment security data. 5HTTLPR genotype results for the 220 adolescents with attachment security data are shown in Table 1. Of these 220 adolescents, 185 (89 males, 96 females; 97 intervention, 88 control) had mothers for whom 5HTTLPR genotype data were available. All analyses were performed on these 220 adolescents and 185 adolescent–mother pairs. No individuals changed from the intervention to the control arm or vice versa at any point in the trial, and no individuals with both genotype and attachment security data were excluded from analysis. The 220 participants with both genotype and attachment data were compared to the rest of the original sample of 449 on a range of demographic and socioeconomic variables. As shown in Table 2, with two exceptions—water and electricity in the home—there were no significant differences between the two groups (See S3 Table for actual values). There were no significant differences on any of the variables between the 110 adolescent participants in the intervention and control groups (S4 Table).

**Table 1:** 5HTTLPR genotype results for the 220 adolescents with both genotype and attachment data, half of whom received the intervention and half of whom did not.

5HTTLPR genotype	Intervention	Control	*N* (%)
Long/long	66	65	131 (60%)
Short/long	39	35	74 (33%)
Short/short	5	10	15 (7%)
**Total**	**110**	**110**	**220**

5HTTLPR, *5HTT-*linked polymorphic region.

**Table 2:** Comparison between the sample of 220 adolescents included in the study and the rest of the original sample of 449.

Covariate	Difference between participants lost to follow-up versus retained	*p*-Value	G×I odds ratio with covariate	95% CI	*p*-Value
Group (intervention or control)	χ^2^ = 0.173	0.706			
Attachment security	χ^2^ = 1.898	0.216			
5HTTLPR	χ^2^ = 0.042	0.882			
No covariate			4.07	1.16, 14.12	0.028
Sex	χ^2^ = 0.219	0.697	4.16	1.19, 14.58	0.026
Housing type	χ^2^ = 0.336	0.593	4.02	1.14, 14.13	0.030
Formal employment	χ^2^ = 1.970	0.195	4.13	1.18, 14.46	0.026
Education	*t* = −0.346	0.729	4.08	1.16, 14.38	0.029
Running water	χ^2^ = 7.618	0.007	3.91	1.11, 13.70	0.033
Electricity	χ^2^ = 4.337	0.038	4.03	1.15, 14.12	0.029

Comparison of group, attachment security, genotype, and demographic variables between the sample of 220 adolescents included in the study and the rest of the original sample of 449 recruited during pregnancy between April 1999 and February 2003 who were lost to follow-up (91), had died (24), or were followed up at adolescence but did not have both attachment and 5HTTLPR genotype data (114), making a total of 229 not being included in this study. All demographic information refers to the mothers at the time of antenatal recruitment. Although two variables (household water and electricity) were significantly different between those included and those not included in the present study, no variable had any appreciable covariate effect on the main G×I result.

5HTTLPR, *5HTT-*linked polymorphic region; G×I, gene × intervention.

### Gene × intervention interaction

**Fig 2:**
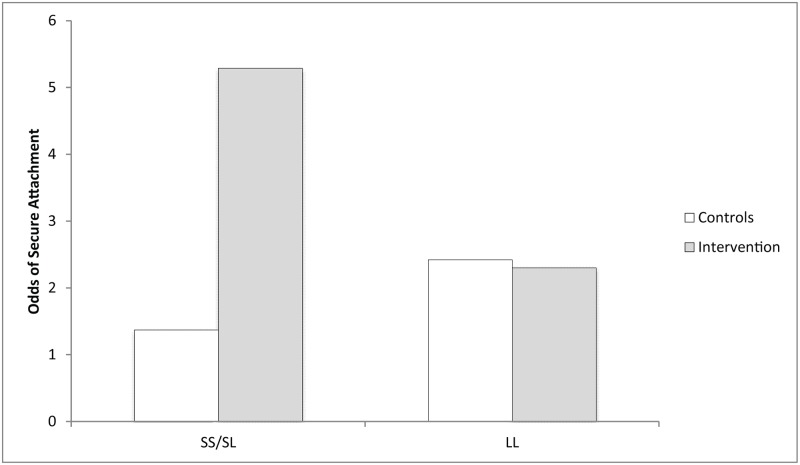
Odds of secure attachment when the 5HTTLPR genotype × home-visiting intervention interaction is taken into account. 5HTTLPR, *5HTT-*linked polymorphic region; LL, long/long; SS/SL, short/short or short/long.

**Fig 3:**
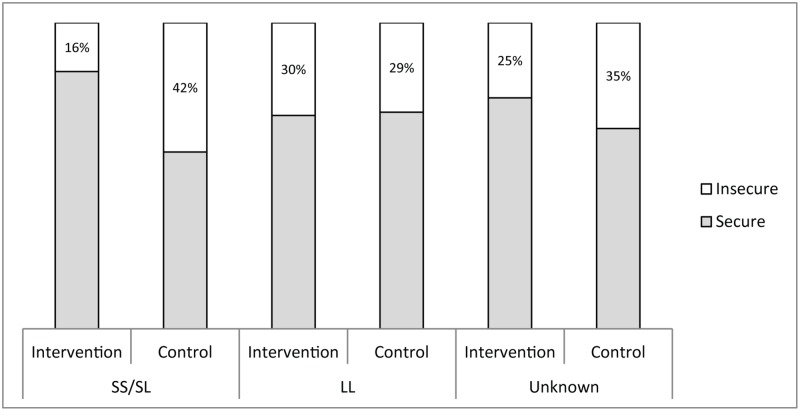
Efficacy of the home-visiting intervention when 5HTTLPR genotype is and is not taken into account. 5HTTLPR, *5HTT-*linked polymorphic region; LL, long/long; SS/SL, short/short or short/long.

Because the presence of at least one short 5HTTLPR allele frequently confers susceptibility to environmental influence [11,17], individuals with short/long and short/short genotypes were treated as one genotype category. Individuals carrying at least one short allele comprised 40% of the 220 participants (Table 1). Logistic regression revealed a significant G×I interaction: for infant security of attachment, the efficacy of the intervention varied as a function of serotonin transporter genotype (OR = 4.07, *p* = 0.028, 95% CI [1.16, 14.20]). As shown in Table 3 and in Fig 2, for those with the susceptible genotype (short/long and short/short), the intervention increased the odds of secure infant attachment nearly 4-fold relative to controls (OR = 3.86, *p* = 0.008, 95% CI [1.42, 10.51], *d* = 0.75). By contrast, for those with the nonsusceptible genotype (long/long), the intervention had no impact on the odds of secure attachment relative to controls (OR = 0.95, *p* = 0.89, 95% CI [0.45, 2.01], *d* = 0.03). Expressed in terms of absolute risk, for those with the short allele, the probability of secure attachment being observed in the intervention group was 84% (95% CI [73%, 95%]), compared to 58% (95% CI [43%, 72%]) in the control group. For those with two copies of the long allele, the probability of being secure was 70% (95% CI [59%, 81%]) in the intervention group, compared to 71% (95% CI [60%, 82%]) in the control group (Table 4; Fig 3). The results show that, on average, individuals carrying at least one short allele were susceptible to the intervention and those carrying two long alleles were nonsusceptible.

**Table 3:** Odds of secure attachment when the 5HTTLPR genotype × home-visiting intervention interaction is taken into account.

5HTTLPR genotype	Odds of secure attachment		Intervention versus control: odds ratio of secure attachment
	Intervention	Control	
Short/short or short/long	5.29	1.37	3.86
Long/long	2.30	2.42	0.95

The data in columns 2 and 3 were used in the calculations depicted in Fig 2. The effect size for the interaction between group and intervention (intervention versus control) is expressed as the odds ratio for secure attachment for each genotype in column 4.

5HTTLPR, *5HTT-*linked polymorphic region.

**Table 4:** Attachment outcomes for each genotype in the intervention and control groups.

Group	5HTTLPR genotype	Number of participants	
		Secure	Insecure
**Intervention**	SS/SL	37	7
	LL	46	20
**Control**	SS/SL	26	19
	LL	46	19

These data are shown in Fig 3 as percentages secure and insecure for each genotype and for all genotypes.

5HTTLPR, *5HTT-*linked polymorphic region.

The efficacy of the home-visiting intervention on the attachment outcome in terms of percentage secure and insecure individuals according to group and genotype is shown in Fig 3. From left to right in Fig 3, for individuals carrying at least one short allele, the percentage showing secure attachment was 84% and 58% in the intervention and control groups, respectively. For individuals carrying two long alleles, the percentage showing secure attachment was 71% and 70% in the intervention and control groups, respectively. In the absence of genetic information, when results are averaged over all individuals and all genotypes (“unknown”), the *apparent* percentage of individuals showing secure attachment was 75% and 65% in the intervention and control groups, respectively. The numbers of individuals in each group and in each genotype category are given in Table 1.

### Sensitivity analyses

The above logistic regression analysis was rerun controlling for covariates observed to be different between the two groups. The result was not affected by sex or any other covariates (Table 2).

Further, to address the possibility that the observed interaction effect was attributable to maternal rather than child genotype, we reran the logistic regression including terms for maternal 5HTTLPR genotype and the interaction between maternal 5HTTLPR and group (intervention versus control) in the model. The child 5HTTLPR × group interaction remained significant (OR = 4.8, *p* = 0.041), while neither the main effect of maternal 5HTTLPR (OR = 0.96, *p* = 0.93) nor its interaction with group (OR = 1.97, *p* = 0.37) was significant.

Finally, multiple imputation analyses based on 100 imputed samples of *n* = 499 (the total number of mother–infant dyads originally randomized) were also run to check the robustness of the result. These analyses confirmed the 5HTTLPR × group interaction in the logistic regression analysis, with the interaction OR equal to −1.41 (standard error = 0.64, *p* = 0.029, 95% CI [−2.68, −0.015]).

## Discussion

The current study aimed to test the hypothesis that 5HTTLPR genotype would moderate the impact of an early child development intervention aimed at promoting the security of mother–infant attachment in a middle-income country. Our reanalysis of the original trial in light of recently acquired genetic information provided support for this hypothesis. Specifically, for children with one or two copies of the short allele of 5HTTLPR, the intervention appeared to be highly effective in improving the rate of attachment security (from 58% in the control group to 84% in the intervention group), but for those with only the long allele, the intervention led to no measurable benefits (secure attachment rate 70% in the control group versus 71% in the intervention group).

There are few studies, and none outside the US and Europe, that have used the framework of experimental trials to test for G×E interaction in early child development [9]. In the specific area of attachment, which is a key domain of psychosocial functioning among young children, we are aware of only two other studies of G×I interaction. In a study of maltreated children who were randomized either to a parenting intervention or to a control condition, Cicchetti and colleagues [13] found no G×I interaction for 5HTTLPR in relation to attachment. There are, however, important differences between this study and our own that may account for the difference in findings. First, the Cicchetti et al. study involved a smaller sample size (in total, ignoring genotype: 49 intervention, 47 control) than our study (110 intervention, 110 control). Second, their sample was racially and ethnically diverse, with the frequency of short allele carriers differing markedly between the black (45.9%), white (78.6%), and other/multiracial (67.5%) categories. By contrast, our study sample was drawn from an ethnically homogeneous population. Third, maltreated children represent a special population that is not comparable to the community sample included in the Thula Sana study. In that regard, it is notable that the disorganized class of insecure attachments, which carries the highest clinical risk, was present in 88% of the maltreated sample and in only 8% of the Thula Sana sample, a figure typical of community samples. Given these important sample differences, little can be concluded from the difference in G×I findings between the two studies. The only other study in this area also relied on a special population sample, in this case, children raised in Romanian institutions. The Bucharest Early Intervention Project randomly allocated institutionalized children to either high-quality foster care or continuing institutional care before 30 mo of age. At 54 mo of age, among children carrying the short/short 5HTTLPR genotype, relative to the outcome of those in the continuing institutional care group, those provided with high-quality foster care had lower symptom levels of attachment disorder (specifically “indiscriminate social behavior”). For the children with at least one long allele (short/long and long/long), there was no difference in terms of attachment disorder symptoms between foster and institutional care conditions [14].

As Belsky recently observed, attachment research, like much research on early child development, has proceeded for the most part with the assumption that all children are equally susceptible to the effects of sensitive and insensitive care [41]. The current findings suggest otherwise and highlight the significance of genetic differential susceptibility in shaping developmental trajectories during early infancy.

An important limitation of this study is that we were not able to follow up all of the individuals from the original trial, and there were missing data for attachment and genotype. In total, our primary analysis included 49% (220/449) of the original sample of children whose mothers were randomized to intervention and control conditions. Although the intervention and control groups were highly similar in our follow-up sample, and the follow-up sample was generally very similar to the original sample, there was some evidence of selective loss to follow-up on two variables (Table 2). This means that randomization within our follow-up sample may have been imperfect. Attribution of the primary outcome to causal effects of the intervention in the present sample should therefore be treated with caution.

Another limitation of this study is its focus on only one gene. Despite extensive evidence from research using both observational and experimental approaches showing that the *5HTT* promoter polymorphism influences organisms’ sensitivity to environmental influences [42], and despite evidence that 5HTTLPR influences the development of functional and structural brain networks involved in emotion regulation, stress processing, and threat sensitivity [43], it is unlikely that one single gene will explain all individual differences in intervention efficacy. Rather, it can be assumed that differential susceptibility to environmental influences is a complex, polygenic trait influenced by the combination of hundreds of common genetic variants of small effect. The first studies in the field of therapygenetics have started using genome-wide approaches [44] and polygenic scoring [45], which allow researchers to aggregate the effects of multiple variants. The use of these approaches in sufficiently large samples, much larger than the present study, could open new avenues in G×I interaction research.

A final limitation is that our attachment finding could be culturally-specific and therefore not generalizable, and certainly the study needs replicating in other cultural contexts. For example, in contexts such as the one described here, where there are multiple caregivers, infants and children are able to develop attachments to more than one person, and when the attachment status is discordant between different caregivers, it remains unclear what the longer-term outcomes are [46]. Nevertheless, we believe it is unlikely that our results would be confined to this cultural context. First, both this sample and one we previously studied in Khayelitsha [24] showed a distribution of attachment categories similar to that in high-income countries. Second, our previous research showed the same association between attachment security and the main parenting antecedents that we would expect (i.e., sensitivity and lack of intrusiveness) from a substantive body of research globally [24].

Beyond illuminating the role of genetic differential susceptibility in early childhood development, the current finding also speaks to a fundamental issue in the quest to understand and mitigate the developmental effects of poverty through psychosocial intervention. The near-large effect size reported here for the intervention in children with susceptible genotypes (*d* = 0.75) is at variance with the general conclusion that psychosocial interventions in the context of poverty produce only small to medium effect sizes [4]. Without taking account of genetic susceptibility, it is possible that other intervention studies have, at least in some subpopulations, underestimated the impact of their interventions, as we originally did. By the same token, as was originally reported for Thula Sana [26], other studies might also have underestimated the negative impact on susceptible subpopulations of *not* receiving an intervention (Figs 2 and 3). In short, averaging outcomes across all participants may well lead to an invalid conclusion about the efficacy of an intervention [47].

The launch of the SDGs and the Global Strategy for Women’s, Children’s and Adolescents’ Health in late 2015 has focused attention on a life-course perspective towards the understanding of child and adolescent development—the “thrive agenda” [48,49]. To stand a chance of meeting the ambitious SDGs and Global Strategy targets by 2030, an enhanced understanding will be required of the biological and psychological mechanisms underlying interventions aimed at improving the lives of young children. In the context of the resource constraints that characterize LMICs, ensuring that psychosocial interventions are implemented in the most efficacious manner will take on an added urgency. In this regard, it is instructive to note a parallel between genetic differential susceptibility to psychosocial interventions and genetic differential susceptibility in the emergence of personalized medicine, specifically pharmacogenomics. Just as genetic information is being used to guide the choice of medication for different individuals diagnosed with the same condition (e.g., [50]), it has been suggested that in a world of limited resources, psychosocial interventions could, once more is known, also be selectively targeted at genetically susceptible individuals [41,51]. This possibility would precipitate the daunting moral challenge of balancing equity (equal treatment for all) and efficacy (treating only those likely to benefit) [41,47].

However, while such targeting in LMICs is technically feasible, provision of intervention services on the basis of genotyping is not currently a realistic prospect. First, as already noted above, genetic prediction of intervention efficacy based on variation at one gene locus is far from sufficiently sensitive or specific to provide a reliable basis for intervention recommendations. Second, quite apart from the science, the prospect of discriminating individuals on the basis of their genetic makeup is controversial and likely to encounter strenuous social resistance.

Nevertheless, other avenues of investigation do suggest themselves. A promising approach might be to incorporate intermediate phenotypes, such as easily accessible physiological or temperamental characteristics, with genetic and epigenetic markers [52] to improve prediction by use of multiple data types. Physiological measures might include hormonal and/or sympathetic and parasympathetic nervous system stress markers [53]; relevant temperamental characteristics could include emotion regulation abilities [54] or approach avoidance tendencies [55]. Indeed, the short/long 5HTTLPR polymorphism has been associated with individual differences in epigenetic methylation [56], stress physiology [57–59], and temperament [60,61]. A combination of biological and behavioral markers could be used to identify meaningful subgroups and thus target interventions to those likely to respond. Moreover, such measures could be used not only to better target interventions to those likely to respond, but also to clarify where new or additional interventions are required.

In summary, despite a considerable body of evidence on how cumulative risk is implicated in poor child development, our understanding of pathways and mechanisms, and how dose, timing, and adversity impact on outcome, is to date quite limited [62]. Measuring genetic susceptibility together with epigenetic, physiological, temperamental, and behavioral markers in RCTs will allow better examination and greater insight into these mechanisms and pathways in LMICs. This could enhance our understanding of why certain individuals do not respond to a particular treatment and facilitate the development of new interventions for them.

## Supporting information

S1 Data Data used for testing main hypothesis and effects of covariates. (XLSX)

S2 Data Data used for multiple imputation analysis of the effects of missing data. (XLSX)

S1 Fig Multiply imputed model parameter estimates by iteration number. (DOCX)

S1 Table Results of multiple imputation logistic regression analysis of intervention group × 5HTTLPR interaction in relation to attachment security. (DOCX)

S2 Table Results of multiple imputation logistic regression analysis of intervention group × 5HTTLPR interaction in relation to attachment security, controlling for maternal genotype and maternal genotype × intervention group interaction. (DOCX)

S3 Table Values for attachment security and demographic variables in the sample of 220 adolescents included in the study compared to the rest of the original sample of 449 who were lost to follow-up (91), had died (24), or were followed up at adolescence but did not have both attachment and 5HTTLPR genotype data (114), making a total of 229 who were not included in this study. Statistical comparisons are given in Table 2.
(DOCX)

S4 Table Comparison between the intervention and control groups for attachment security, genotype, and demographic variables. No significant differences were found.
(DOCX)

S1 Text Details of the multiple imputation analyses testing for the effect of missing data on the main hypothesis. (DOCX)

S2 Text Prespecified protocol. (DOCX)

## Abbreviations

5HTTLPR: *5HTT-*linked polymorphic region
G×E: gene × environment
G×I: gene × intervention
LMICs: low- and middle-income countries
OR: odds ratio
RCT: randomized controlled trial
SDG: Sustainable Development Goal
SSP: strange situation procedure
