# Supplementary material for: Serotonin transporter gene (SLC6A4) polymorphism and susceptibility to a home-visiting maternal-infant attachment intervention delivered by community health workers in South Africa: Reanalysis of a randomized controlled trial
Source: PLoS Med. 2017 Feb 28;14(2):e1002237. doi: 10.1371/journal.pmed.1002237 (PMC5330451; doi:10.1371/journal.pmed.1002237)
Supplement: S1 Table — (DOCX) [file pmed.1002237.s004.docx]

|  | B | Std. Err. | t | p | 95% CI | |
| --- | --- | --- | --- | --- | --- | --- |
|  |  |  |  |  |  |  |
| 5HTTLPR | 0.87 | 0.47 | 1.86 | 0.06 | -0.05 | 1.80 |
| Group | 1.37 | 0.48 | 2.85 | 0.01 | 0.43 | 2.31 |
| Interaction | -1.41 | 0.64 | -2.20 | 0.03 | -2.68 | -0.15 |
| Date | 5.9^-4^ | 6.1^-4^ | -0.98 | 0.33 | 1.8^-3^ | 6.0^-4^ |
| Water | 0.37 | 0.45 | 0.82 | 0.41 | -0.51 | 1.25 |
| Electricity | -0.49 | 0.44 | -1.13 | 0.26 | -1.35 | 0.36 |
| Constant | 7.31 | 9.07 | 0.81 | 0.42 | -10.48 | 25.09 |

**Table S1.** Results of multiple imputation logistic regression analysis of group x 5HTTLPR interaction in relation to attachment security.
