# Supplementary material for: Serotonin transporter gene (SLC6A4) polymorphism and susceptibility to a home-visiting maternal-infant attachment intervention delivered by community health workers in South Africa: Reanalysis of a randomized controlled trial
Source: PLoS Med. 2017 Feb 28;14(2):e1002237. doi: 10.1371/journal.pmed.1002237 (PMC5330451; doi:10.1371/journal.pmed.1002237)
Supplement: S2 Table — (DOCX) [file pmed.1002237.s005.docx]

|  | B | Std. Err. | t | p | 95% CI | |
| --- | --- | --- | --- | --- | --- | --- |
|  |  |  |  |  |  |  |
| Child 5HTTLPR | 1.18 | 0.54 | 2.20 | 0.03 | 0.12 | 2.24 |
| Maternal 5HTTLPR | 0.56 | 0.53 | 1.06 | 0.29 | -0.48 | 1.61 |
| Group | 1.93 | 0.71 | 2.72 | 0.01 | 0.53 | 3.32 |
| Child 5HTTLPR x group | -1.83 | 0.72 | -2.55 | 0.01 | -3.24 | -0.42 |
| Maternal 5HTTLPR x group | -0.73 | 0.75 | -0.98 | 0.33 | -2.20 | 0.74 |
| Date | 6.0^-4^ | 6.4^-4^ | -0.94 | 0.35 | 1.9^-3^ | 6.5^-4^ |
| Water | 0.40 | 0.45 | 0.89 | 0.37 | -0.48 | 1.27 |
| Electricity | -0.52 | 0.44 | -1.16 | 0.24 | -1.38 | 0.35 |
| Constant | 6.95 | 9.57 | 0.73 | 0.47 | -11.83 | 25.72 |

**Table S2.** Results of multiple imputation logistic regression analysis of group x 5HTTLPR interaction in relation to attachment security, controlling for maternal genotype and maternal genotype x group interaction.
