# Supplementary material for: Serotonin transporter gene (SLC6A4) polymorphism and susceptibility to a home-visiting maternal-infant attachment intervention delivered by community health workers in South Africa: Reanalysis of a randomized controlled trial
Source: PLoS Med. 2017 Feb 28;14(2):e1002237. doi: 10.1371/journal.pmed.1002237 (PMC5330451; doi:10.1371/journal.pmed.1002237)
Supplement: S3 Table — Statistical comparisons are given in Table 2. (DOCX) [file pmed.1002237.s006.docx]

**Table S3.** Values for attachment security and demographic variables in the sample of 220 adolescents included in the study compared to the rest of the original sample of 449 who were either lost to follow-up (91), had died (24), or who were followed up at adolescence but did not have both attachment and 5HTTLPR genotype data (114), making a total of 229 being not included in this study. Statistical comparisons are given in Table 2.

|  | N  included  /not included | Units | Included | Not included |
| --- | --- | --- | --- | --- |
| Group | 220/229 | Participants | 220 | 229 |
| Attachment security | 220/45 | secure/insecure | 155/65 | 27/18 |
| 5HTTLPR | 220/59 | SS & SL/LL | 89/131 | 23/36 |
| Sex | 220/204 | male/female | 105/115 | 102/102 |
| Housing type | 218/227 | Formal/informal | 184/34 | 196/31 |
| Employment | 220/227 | Yes/no | 25/195 | 17/210 |
| Education | 220/227 | Mean (std dev) | 8.48 (8.94) | 7.87 (6.42) |
| Running water | 217/226 | Yes/no | 133/84 | 109/117 |
| Electricity | 220/228 | Yes/no | 122/98 | 104/124 |
