# Supplementary material for: Serotonin transporter gene (SLC6A4) polymorphism and susceptibility to a home-visiting maternal-infant attachment intervention delivered by community health workers in South Africa: Reanalysis of a randomized controlled trial
Source: PLoS Med. 2017 Feb 28;14(2):e1002237. doi: 10.1371/journal.pmed.1002237 (PMC5330451; doi:10.1371/journal.pmed.1002237)
Supplement: S4 Table — No significant differences were found. (DOCX) [file pmed.1002237.s007.docx]

**Table S4.** Comparison between the intervention and control groups for attachment security, genotype and demographic variables. No significant differences were found.

|  | N intervention  /control | Group comparison | P value | Units | Intervention | Control |
| --- | --- | --- | --- | --- | --- | --- |
| Attachment security | 110/110 | $\chi$*^2=^*2.64 | 0.139 | secure/insecure | 83/27 | 72/38 |
| 5HTTLPR | 110/110 | $\chi$*^2=^*0.19 | 1.000 | SS & SL/LL | 44/66 | 45/65 |
| Sex | 110/110 | $\chi$*^2=^*0.164 | 0.787 | male/female | 51/59 | 54/56 |
| Housing type | 109/109 | $\chi$*^2=^*1.25 | 0.354 | Formal/informal | 20/89 | 14/95 |
| Employment | 110/110 | $\chi$*^2=^*0.406 | 0.672 | Yes/no | 14/96 | 11/99 |
| Education | 110/110 | t=0.346 | 0.729 | Mean (std dev) | 8.69 (8.9) | 8.27 (9.0) |
| Running water | 107/110 | $\chi$*^2=^*0.26 | 0.890 | Yes/no | 65/42 | 68/42 |
| Electricity | 110/110 | $\chi$*^2=^*0.294 | 0.684 | Yes/no | 59/51 | 47/63 |
