# Supplementary material for: Serotonin transporter gene (SLC6A4) polymorphism and susceptibility to a home-visiting maternal-infant attachment intervention delivered by community health workers in South Africa: Reanalysis of a randomized controlled trial
Source: PLoS Med. 2017 Feb 28;14(2):e1002237. doi: 10.1371/journal.pmed.1002237 (PMC5330451; doi:10.1371/journal.pmed.1002237)
Supplement: S1 Text — (DOCX) [file pmed.1002237.s008.docx]

**S1 Text**

Details of the multiple imputation analyses testing for the effect of missing data on the main hypothesis.

*Multiple imputation*

Due to the presence of missing data our primary hypotheses were re-tested using multiple imputation, which under a range of circumstances provides less biased estimates of model parameters than listwise deletion when data are missing at random (MAR). There are a range of methods for conducting multiple imputation (MI) that are suitable for different circumstances. We used the Fully Conditional Specification approach, which is a highly flexible approach capable of accounting for non-linearity in the relationship between covariates and outcome. The analyses reported in this paper were non-linear in two senses – they relied on a logistic regression model and tested covariate-by-covariate interaction (group x 5HTTLPR genotype). In such circumstances, failing to take account of the presumed non-linearity of these relationships can lead to bias in standard MI approaches because the MI generating model may be incompatible with the substantive model under investigation. The FCS method developed by Bartlett and colleagues (2014) takes account of the substantive model in generating the imputation samples. Full technical details of the procedure are given in Bartlett et al. (2014). We used the STATA command *smcfcs* for these analyses (Bartlett & Morris, 2015). In addition to the variables used in our substantive analyses (group, 5HTTLPR and their interaction) we included two covariates identified as different between the initially randomized sample and the follow-up sample (family home having piped water, and electricity), as well as date of entry into the study. A burn-in of 10 iterations was used, and 100 imputation samples were generated. We tested for convergence by examining model parameters over 1000 iterations. The imputed samples consisted of all 449 cases originally randomized into the trial. These plots for our primary parameter estimates of interest (group, 5HTTLPR, their interaction and the model constant) are shown in S1 Fig. The plots suggested reasonable evidence of convergence (no long-term drift or marked changes in variance).

The results of the multiple imputation analysis are summarised in S1 Table.

As S1 Table indicates, accounting for missing data using FCS-MI did not lead to any marked changes in the parameter estimate for the interaction between group and 5HTTLPR genotype (B = -1.41, 95% CI[-2.68, -.15].

The smcfcs model command used was as follows:

**smcfcs logistic secure cgene group cint date elec, logit(cgene water elec) passive(cint = cgene*group) m (100)**

Similarly, we re-ran the model that included both child and maternal genotype under FCS-MI using the same additional covariates. The results are shown in Table S2.

As Table S2 indicates, the group x 5HTTLPR interaction remained substantively unchanged after maternal genotype and the maternal genotype x group interaction were controlled for in the multiple imputation analysis.

The *smcfcs* model command for this analysis was:

**smcfcs logistic secure cgene mgene group cint mint date water elec, logit(cgene mgene water elec ) passive(cint = cgene*group| mint=mgene*group) m(100)**

**References**

Bartlett, J. W., & Morris, T. P. (2015). Multiple imputation of covariates by substantive-model compatible fully conditional specification. *The Stata Journal*, *15*(2), 437-456.

Bartlett, J. W., Seaman, S. R., White, I. R., Carpenter, J. R., & Alzheimer's Disease Neuroimaging Initiative. (2015). Multiple imputation of covariates by fully conditional specification: accommodating the substantive model. *Statistical Methods in Medical Research*, *24*(4), 462-487.
