# Supplementary material for: Serotonin transporter gene (SLC6A4) polymorphism and susceptibility to a home-visiting maternal-infant attachment intervention delivered by community health workers in South Africa: Reanalysis of a randomized controlled trial
Source: PLoS Med. 2017 Feb 28;14(2):e1002237. doi: 10.1371/journal.pmed.1002237 (PMC5330451; doi:10.1371/journal.pmed.1002237)
Supplement: S2 Text — (DOCX) [file pmed.1002237.s009.docx]

S2 Text

Documentary evidence of the a priori plan to investigate the 5HTTLPR as the primary hypothesis in this study. Below are excerpts from a funding proposal submitted to the *South African Medical Research Council* in 2012.

**South African Medical Research Council**

**Self-Initiated Research Proposal Form**

**Please e-mail completed form to:** [**rgmd@mrc.ac.za**](mailto:rgmd@mrc.ac.za)

**Questions and clarifications can be directed to Tania Brodocvky (**[**taniab@sun.ac.za**](mailto:taniab@sun.ac.za)**) or Clive Glass at 021-938-0225**

**Summary** This proposal aims to add genetic data to a recently well-funded interdisciplinary study investigating long-term psychosocial and psychophysiological effects in children whose pregnant mothers participated in a randomised controlled intervention trial encouraging sensitive, responsive mothering 13 years ago. Initial follow-up found significantly higher maternal sensitivity and a higher rate of secure infant attachment at 18 months. The intervention was also beneficial in terms of maternal mood and infant cognitive outcome. New funding (>R5m) has recently been acquired from Grand Challenges Canada to assess cognitive, emotional, psychophysiological, neuroendocrine, psychological and psychosocial variables in the children who are now 13 years old. A limitation of the original study is the absence of information regarding genetic differences known to moderate the effects of early parenting experiences. Polymorphisms in so-called ‘susceptibility to parenting’ genes are known to interact with early parenting styles in ways that moderate the main outcome measures used to assess the effects of the original intervention. The absence of such genetic information thus fails to make the most of a range of new high quality data being collected in the current follow-up study of the same children who are now 13 years of age. This proposal therefore seeks additional funding for genetic studies that will add significant value to the research as a whole, including retrospective re-analysis of the data collected when the children were between 6 and18 months old.

Excerpt page 15

*Treatment of Primary and Secondary Gene Candidates*

These gene candidates thus code for, or regulate the expression of proteins intimately involved in the stress response and represent strong candidates for genetic moderators of environmental stressors. However, less is known directly about their involvement in G x E either for allostatic load or for psychopathology more globally. **We thus propose to investigate the 5HTTLPR polymorphism as our primary candidate for G x E in adjustment and allostatic load, and treat the additional genes described above as secondary candidates. In the former case, our statistical analyses will be uncorrected for type I error in order to maximize statistical power, while the latter genes will be tested against stringent bonferroni corrected alpha levels.**
